# The effects of anticholinergic medications on cognition in children: a systematic review and meta-analysis

**DOI:** 10.1038/s41598-020-80211-6

**Published:** 2021-01-08

**Authors:** Erica Ghezzi, Michelle Chan, Lisa M. Kalisch Ellett, Tyler J. Ross, Kathryn Richardson, Jun Ni Ho, Dayna Copley, Claire Steele, Hannah A. D. Keage

**Affiliations:** 1 Justice and Society, University of South Australia, GPO BOX 2741, Adelaide, 5000 Australia; 2 Clinical and Health Sciences, University of South Australia, Adelaide, Australia; 3 Norwich Medical School, University of East Anglia, Norwich, UK

**Keywords:** Outcomes research, Cognitive ageing, Paediatric research

## Abstract

Cognitive side effects of anticholinergic medications in older adults are well documented. Whether these poor cognitive outcomes are observed in children has not been systematically investigated. We aimed to conduct a systematic review and meta-analysis on the associations between anticholinergic medication use and cognitive performance in children. Systematic review was conducted using Medline, PsychInfo, and Embase, identifying studies testing cognitive performance relative to the presence versus absence of anticholinergic medication(s) in children. We assessed effects overall, as well as relative to drug class, potency (low and high), cognitive domain, and duration of administration. The systematic search identified 46 articles suitable for meta-analysis. For the most part, random effects meta-analyses did not identify statistically significant associations between anticholinergic exposure and cognitive performance in children; the one exception was a small effect of anticholinergic anti-depressants being associated with better cognitive function (Hedges’ *g* = 0.24, 95% CI 0.06–0.42, *p* = 0.01). Anticholinergic medications do not appear to be associated with poor cognitive outcomes in children, as they do in older adults. The discrepancy in findings with older adults may be due to shorter durations of exposure in children, differences in study design (predominantly experimental studies in children rather than predominantly epidemiological in older adults), biological ageing (e.g. blood brain barrier integrity), along with less residual confounding due to minimal polypharmacy and comorbidity in children.

## Introduction

Anticholinergic medications are commonly prescribed^1–3^ yet a growing body of evidence has demonstrated that their use is associated with a higher risk of incident cognitive impairment^4–6^. This literature has been reviewed multiple times in older adults, whereby anticholinergic medications have been consistently associated with cognitive decline and dementia^7–9^. There has been no systematic synthesis of the cognitive effects of anticholinergic medications in children.

There are few population-based studies that have assessed the extent to which children are exposed to anticholinergic medicines^1^. Most studies examining anticholinergic medicines in children have focussed on the use of medicine classes for specific indications, for example, asthma or overactive bladder, rather than providing population-based estimates for the use of anticholinergic medicines like the studies in older adults. Approximately 11% of Australian children have a current diagnosis of asthma^10^ and up to 20% of children experience bedwetting^11^ so there is potential for a high prevalence of use of anticholinergic medicines to treat these conditions in children. One population based study from Slovenia reported that 20% of children using prescription medicines were dispensed anticholinergic medicines, most commonly antihistamines^1^.

Anticholinergic medications refer to a broad class of medicines which block the neurotransmitter acetylcholine^12^. These medications are used in the treatment of many conditions such as depression, vertigo, asthma, cardiac arrhythmias and incontinence. High potency anticholinergic medications appear to most detrimentally affect cognition in older adults (as compared to low potency)^13^. Further, the class of anticholinergic medication differentially associates with cognitive decline in late-life, with anti-depressants (amitriptyline, dosulepin, paroxetine), urologicals (oxybutynin, tolterodine), and antiparkinsonian drugs showing the strongest associations with incident dementia^4^. Neurobiologically, the cholinergic system primarily mediates attentional processes^14–17^ and therefore could be expected to be primarily impaired by anticholinergic medications, although cognitive domain specific effects have not been investigated.

The current study aims to quantitively synthesise the literature on associations between anticholinergic medications and cognitive performance in children. Findings from this review will inform medical practitioners of any risks (or lack thereof) associated with anticholinergic use in children, and subsequently help to inform the safe prescribing of anticholinergics. It is critical to identify whether anticholinergics should be prescribed with restraint in children. We hypothesise that in children (1) exposure to anticholinergic medications will be significantly negatively associated with performance on cognitive tests, and that associations will be strongest for (2) antidepressant and urological drug classes (as compared to other drug classes), (3) high-potency anticholinergics (as compared to low-potency), (4) those exposed long-term (as opposed to short-term) and (5), within the cognitive domain of attention.

## Methods

### Search strategy

This study adhered to the Preferred Reporting Items for Systematic Reviews and Meta-Analyses (PRISMA) guidelines (see Supplementary Table 1 for PRISMA Checklist)^18,19^. A systematic literature search was conducted in December 2019 using the electronic databases Medline, PsychInfo, and Embase. The search strategy used a combination of keywords for anticholinergic medications (see Supplementary Material), cognition terms (cognit* OR neuropsych* OR learn* OR memory OR "executive function" OR "executive functions") and demographic terms (children OR childhood OR youth* OR teen*). No published review protocol exists for the current study.

Anticholinergic medications were defined as medicines with clinically significant anticholinergic properties as listed in a systematic review by Duran et al.^12^. Medications assessed by the Duran systematic review to be of either high or low anticholinergic potency, but not ambiguous potency, were included. Studies were screened and assessed for eligibility by two independent reviewers, first by title and abstract, then by full text, according to inclusion and exclusion criteria described below (MC, TJR, DC, CS and JNH). Any conflicts were resolved through consensus.

### Inclusion and exclusion criteria

Studies of either within- or between-groups design were included if they reported at least one cognitive outcome for both children exposed and unexposed to anticholinergic medications; reported data for a sample of children (< 18 years old); were published in English; and were published in peer-reviewed journal articles. Studies from all publication years were accepted. “On” medication participants included children exposed to at least one anticholinergic medication. “Off” medication participants included matched controls unexposed to any other medication, participants treated with placebo, participants undergoing withdrawal from the medication, or the baseline measurements of the exposed group. To be eligible for inclusion, studies needed to report cognitive outcomes based on objective cognitive measures; subjective behavioural reports were not included (e.g. self, parent or teacher reports of cognitive functioning). Studies were excluded if the control group did not share the same disorder or symptom (i.e. healthy control group) of the experimental group. Studies which only compared the effects of anticholinergic medication versus non-anticholinergic medication, rather than anticholinergic medication versus no medication, were excluded. Studies were also excluded if they involved non-human (animal) participants; if they assessed in-utero anticholinergic exposure; or if they were a case report, case series, thesis or conference abstract.

### Data extraction

Data were extracted from eligible studies independently by one reviewer (EG, MC, TJR) and then checked by a second reviewer, with any discrepancies resolved through discussion or checked again (by a third reviewer). Extracted data include country of publication, study design, sample size (and number of male/female participants), age, diagnoses of sample, name of medication, duration of administration, and cognitive domains assessed. The extracted medication name was then classified by potency and drug class by an academic pharmacist (LE). Data required for meta-analysis were also extracted. This included any data for which an effect size (standardised mean difference) could be calculated for differences between on and off medication groups (e.g., means and standard deviations, Cohen’s d and confidence intervals (CIs), sample size and correlation statistic, means and correlation statistic, or means and p-value).

### Quality assessment

A quality assessment tool was developed for this study, adapted from a critical appraisal tool for randomised controlled trials from the Joanna Briggs Institute^20^, see Supplementary Material—Quality Assessment Tool. The Joanna Briggs Institute is a highly regarded organization with recommended^21^ and well-used critical appraisal checklists^22–24^. The quality assessment tool comprised an eight-point checklist. All studies were screened using this tool by two independent reviewers (MC and TJR) and any conflicts in scoring were resolved through discussion.

### Statistical approach

Some included studies reported data for both within- and between-groups designs. For example, they may include two groups: one that experiences a period of on and off medication, and one non-medicated control group. In these cases, the between-group design (i.e. medication versus control) was preferentially selected in order to minimise the effect of cognitive development (over time). Where one study reported both within- and between-group comparisons for two distinct participant samples (i.e. one group both on and off medication, along with a second group on medication and a third no-medication control group) both within- and between-groups data were extracted. In cases where one study reported both (within and between) comparisons over multiple time-points, within-groups data were extracted for any time-points where between-groups data were unavailable.

All outcome measures were standardised using Hedges’ *g* for difference between on- and off-medication groups. A positive Hedges’ *g* represents a better cognitive score for the on-medication group compared to the off-medication group, regardless of the direction of the original cognitive test. Small, medium, and large effect sizes were classified using the Hedges and Olkin^25^ method, as 0.20, 0.50, and 0.80 respectively. Comprehensive Meta-Analysis software (version 3) was used to calculate effect sizes, where calculations of Hedge’s *g* are dependent on study design (within- or between-groups). Statistical analyses were conducted using the meta package^26^ for R (Version 4.0.2). Dependency was present in analyses due to included studies reporting multiple cognitive outcomes or time-points for follow-up based on the same, or largely overlapping, participant samples. This was accounted for by averaging across effect sizes within studies, so one effect size was used per study within each analysis. The data and script associated with this analysis are publicly available (https://github.com/ericaghezzi/anticholinergic_med_metaanalysis).

Outcomes across studies were pooled using a random-effects model. The commonly used DerSimonian and Laird^27^ estimator of between-study variance has been criticised due to its propensity to underestimate true between-study variance, leading to narrow CIs and potential false-positive estimations^28,29^. Hence, we followed the recommendation of Veroniki et al.^30^ and employed the Paule and Mandel^31^ method, which has been shown to be less biased^29,32^ when estimating between-study variance. Sensitivity analyses revealed no substantial differences in outcomes when analyses were run using common between-groups estimators. The Hartung-Knapp method for random effects meta-analysis^33,34^ was also applied to all analyses. A result was considered statistically significant when *p* < 0.05. We considered this an exploratory study and did not correct for multiple comparisons. Between-study variance was quantified using τ^2^. The proportion of between-study heterogeneity out of total variance was assessed using the I^2^ statistic. Values of I^2^ were classified as low (25%), moderate (50%), or high (75%)^35^.

### Subgroup analysis

Subgroup analyses were stratified by anticholinergic potency, cognitive domain, drug class, and duration of medication administration. Anticholinergic potency was classified as low or high according to Durán et al.^12^. Cognitive domain was based on Lezak et al.^36^: attention, psychomotor functioning, concept formation and reasoning, perception, memory, executive function, language, and intelligence. The anticholinergic drugs administered were categorised by class as antiepileptics (WHO Anatomical Therapeutic Chemical code N03), antiparkinsonian medicines (N04B), antipsychotics (N05A), antidepressants (N06A), respiratory medicines (R), opioid analgesics (N02A), or urological medicines (G04B). Only one study^37^ reported results based on an antiparkinsonian anticholinergic, so subgroup meta-analysis of this medication class was not conducted (note: the study was included in the overall meta-analysis). Total volume of exposure or dose has been shown to be important in assessing risk of cognitive impairment associated with use of anticholinergic medicines in adults; however, dose was inconsistently reported, or not reported at all, in many of the studies included in the meta-analysis. Duration of exposure, which was consistently reported in the studies, was therefore analysed. Duration of medication administration was categorised as either (1) current and long-term (> 1-month), (2) current and acute (≤ 1-month) and (3) historical administration. Each subgroup analysis was based on a random-effects model, where calculations of within-subgroup variance and comparisons between subgroups were both made using a random-effects model. Fixed effects comparisons of differences between subgroups were not made due to the risk of false positives^38^. The Q statistic was calculated as a test of between subgroups differences.

*Publication bias.* Funnel plots of effect size versus standard error for the primary outcome were visually examined for symmetry to assess for bias across studies due to the small-study effect^39^. As the whole meta-analysis contained at least 10 studies, small-study effect was formally tested using Egger’s test of the intercept^40^. If evidence of asymmetry was found (one-tailed *p* < 0.1 on the Egger’s test), Duval and Tweedie’s^41^ trim and fill method would have been used to quantify the magnitude of potential bias.

## Results

### Summary of studies

**Figure 1:**
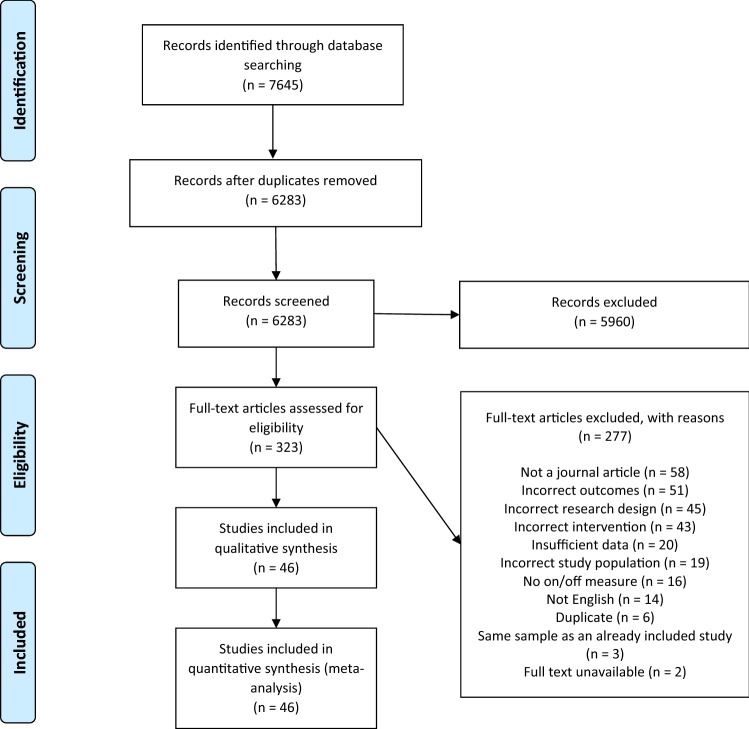
PRISMA flow diagram of the article selection and screening process. The databases searched were Medline, PsychInfo, and Embase.

A total of 7,645 articles were identified, of which 6,283 were screened by title and abstract following duplicate removal. Full-text review was conducted on 323 articles, and 46 of these were included for final review and meta-analysis (Fig. 1). The 46 included studies were published across 6 decades, with 1, 2, 7, 10, 13, and 13 studies published in ascending decades from the 1960s. Of the included studies, 37 were conducted in developed countries, 7 in developing countries, and 2 included children from both developing and developed countries (classified according to the UN^42^). For a complete overview of the characteristics of included studies, see Table 1.

**Table 1:** Demographic, sample, anticholinergic medication and cognitive outcome characteristics for included studies within meta-analysis for cognitive outcomes on and off anticholinergic medication.

Author	Year	Country	Design	Sample			Anticholinergic medication					
				N (M/F)	Age in years*	Diagnoses	Name	Potency	Class/function	Length of administration	Medication duration	Cognitive domain(s)
Aldenkamp et al.^43^	1993	Sweden	NRCT (Within)	83 (47/36)	12.8 (2.4)	Epilepsy	Carbamazepine	Low	Antiepileptic	Long	> 1 year	Att., PM
Aman et al.^44^	2008	USA	RCT (Between**)	38 (29/9)	9.4 (3.0)	Autism + Severe behavioural disturbance	Risperidone	Low	Antipsychotic	Acute, Long	4 weeks, 8 weeks	Att., CF + R, Mem., Perc., PM,
Aman et al.^45^	2009	USA	RCT (Crossover)	16 (14/2)	8.6 (2.6)	DBD/ADHD/High-functioning autism	Risperidone	Low	Antipsychotic	Acute	2 weeks	Att., PM
Barrickman et al.^46^	1991	USA	NRCT (Within)	19 (16/3)	11.0 (2.3)	ADHD	Fluoxetine	Low	Antidepressant	Long	6 weeks	Att., EF, Int
Beers et al.^37^	2005	USA	RCT (Between**)	13	11.9 (3.0)	TBI	Amantadine	Low	Antiparkinsonian	Long	12 weeks	Att., CF + R, EF
Bender and Milgrom^47^	2004	USA	RCT (Between)	60	[8–17]	SAR	Loratadine	Low	Respiratory	Acute	2 weeks	Att., Mem
Bender et al.^48^	1991	USA	NRCT (Between)	63	11.7 (2.1)	Asthma	Theophylline	Low	Respiratory	Acute	1 week, 1 month, 3 months, 6 months	Att
Carlson et al.^49^	1992	USA	NRCT (Crossover)	11 (8/3)	8.7 (2.4)	CD with manic symptoms/CD with family BPD history/Aggressive behaviour	Lithium	Low	Antipsychotic	Acute, Long	4 weeks, 8 weeks	Att., EF + R, Mem
Chen et al.^50^	2001	Taiwan	NRCT (Within)	25 (13/12)	11.2 (2.0)	Epilepsy	Carbamazepine	Low	Antiepileptic	Long	> 1 year	Int
de Graaf et al.^51^	2011	Netherlands	RCT (Between)	90 (51/39)	< 3d at exposure; 5 at follow up	Pain	Morphine	Low	Opioid analgesic	History	NR	Int., PM
de Graaf et al.^52^	2013	Netherlands	RCT (Between)	89 (56/33)	< 3d at exposure; 8 – 9 at follow-up	Pain	Morphine	Low	Opioid analgesic	History	NR	Att., CF + R, EF, Int., PM
Donati et al.^53^	2007	Europe (7 countries)	RCT (Within)	83 (37/46)	10 [6–16]	Partial seizures	Oxcarbazepine, Carbamazepine	Low	Antiepileptic	Long	6 months	Att., Mem, Perc., PM
Erickson et al.^54^	1984	USA	RCT (Within)	11	14.2 (12.9–18.6)	Schizophrenia/Schizophreniform disorder	Thioridazine, Thiothixene	High	Antipsychotic	Long	35 days	Att
Eun et al.^55^	2012a	South Korea	RCT (Within)	41 (24/17)	8.3 (2.1)	Epilepsy	Carbamazepine	Low	Antiepileptic	Long	32 weeks	Int
Eun et al.^56^	2012b	South Korea	NRCT (Within)	168 (98/70)	8.4 (2.7)	Epilepsy	Oxcarbazepine	Low	Antiepileptic	Long	26–32 weeks	Att., Int., CF + R, Lan., PM
Farmer et al.^57^	2017	USA	RCT (Between)	165 (128/3)	8.9 (2.0)	ADHD + Severe physical aggression	Risperidone	Low	Antipsychotic	Acute	3 weeks	Att
Ferguson et al.^58^	2012	USA	RCT (Between)	19 (12/7)	Neonate exposure; 6.2 (0.3) at follow-up	Pain	Morphine	Low	Opioid analgesic	History	≤ 14 days	Att., CF + R, Int., Lan
Forsythe et al. ^59^	1991	UK	RCT (Within)	14 (7/7)	10	Epilepsy	Carbamazepine	Low	Antiepileptic	Acute, Long	1 month, 6 months, 12 months	Att., Mem
Freibergs et al.^60^	1968	Canada	RCT (Between**)	36 (36/0)	8.7 (6–12)	Hyperactivity	Chlorpromazine	High	Antipsychotic	Long	74.8 days	CF + R
Giramonti et al.^61^	2008	USA	RCT (Crossover)	14 (9/5)	7.7 (2.0)	Incontinence	Oxybutynin, Tolterodine	High	Urological	Acute	2 weeks	Att., Mem
Gualtieri and Evans ^62^	1988	USA	RCT (Crossover)	9 (6/3)	9.5 (1.3)	ADHD	Imipramine	High	Antidepressant	Acute	2–3 days	Att., PM
Gualtieri et al.^63^	1991	USA	RCT (Crossover)	12 (11/1)	[6–12]	ADHD	Desipramine	High	Antidepressant	Acute	2–3 days	Att., Mem, PM
Gunther et al.^64^	2006	Germany	NRCT (Within)	23 (21/2)	11.9 (2.1)	ADHD + DBD	Risperidone	Low	Antipsychotic	Acute	4 weeks	Att., EF
Jung et al.^65^	2015	South Korea	RCT (Within)	40	^[4–16]^	Epilepsy	Carbamazepine	Low	Antiepileptic	Long	52 weeks	Int
Klein^66^	1990	USA	RCT (Within & Between)	36 (33/3)	8.5 (1.6)	ADHD + Hyperactivity	Thioridazine	High	Antipsychotic	Acute, Long	4 weeks, 12 weeks	Att., CF + R, EF, Int., Lan., Mem., PM
Kwon et al.^67^	2013	South Korea	NRCT (Between**)	29 (17/15)	8.4 (2.3)	Epilepsy	Oxcarbazepine	Low	Antiepileptic	Long	6 months	Att., CF + R, EF, Int
O'Dougherty et al.^68^	1987	USA	NRCT (Within)	11 (4/7)	9.8 (3.1)	Epilepsy	Carbamazepine	Low	Antiepileptic	Long	3 weeks–10 months	Att., Mem, PM
Operto et al.^69^	2020	Italy	NRCT (Within)	46 (16/20)	9.8 (2.3)	Epilepsy	Oxcarbazepine, Carbamazepine	Low	Antiepileptic	Long	9 months	Comp
Pandina et al.^70^	2009	Europe (6 countries), Israel, South Africa	RCT (Within & Between)	284 (248/36)	10.8 (2.9)	DBD	Risperidone	Low	Antipsychotic	Long	6 weeks, 6 months	Att., Mem
Piccinelli et al.^71^	2010	Italy	NRCT (Within)	43 (21/22)	10.4 (3.1)	Epilepsy	Carbamazepine	Low	Antiepileptic	Long	12 months	CF + R, Int
Platt et al.^72^	1981	USA	RCT (Between**)	30 (28/2)	9.0 (5.8–12.9)	CD	Haloperidol, Lithium	Low	Antipsychotic	Acute	4 weeks	Att., EF
Platt et al.^73^	1984	USA	RCT (Between**)	61 (57/4)	9.0 (5.2–12.9)	CD	Haloperidol, Lithium	Low	Antipsychotic	Acute	4 weeks	Att., EF
Rappaport et al.^74^	1989	USA	RCT (Crossover)	17 (11/6)	[6–12]	Asthma	Theophylline	Low	Respiratory	Acute	3.5 days	Att., EF, Mem., PM
Robles et al.^75^	2011	Spain	RCT (Within)	49 (38/11)	15.9 (1.4)	Psychosis	Quetiapine, Olanzapine	Low	Antipsychotic	Long	6 months	Att., CF + R, Comp., EF, Mem., Perc., PM
Schlieper et al.^76^	1991	Canada	RCT (Crossover)	31 (21/10)	9.8 (1.6)	Asthma	Theophylline	Low	Respiratory	Acute	10 days	Att., EF Mem
Seidel and Mitchell^77^	1999	USA	NRCT (Crossover)	10 (6/4)	9.7 (2.0)	Epilepsy	Carbamazepine	Low	Antiepileptic	Long	2.2 months–2.1 years	Att., CF + R, Int., Lan., Mem., PM
Shehab et al.^78^	2016	Lebanon	NRCT (Within)	24 (8/16)	14.8 (1.6)	MDD	Fluoxetine	Low	Antidepressant	Long	6 weeks, 12 weeks	Att., EF
Sommer et al.^79^	2005	USA	NRCT (Between**)	25 (11/14)	7.2 (1.8)	Incontinence	Oxybutynin	High	Urological	Acute	4 weeks	Att., Mem
Stevenson et al.^80^	2002	Europe (12 countries), Brazil, Canada	RCT (Between)	165	2.92	Dermatitis	Cetrizine	Low	Respiratory	Long	8 weeks	Comp
Tonnby et al.^81^	1994	Sweden	NRCT (Within)	100 (56/44)	12.5 (2.1)	Epilepsy	Carbamazepine	Low	Antiepileptic	Long	Approx. 3.7 years	Att., Mem., PM
Troost et al.^82^	2006	Netherlands	RCT (Within)	24 (22/2)	9.3 (2.6)	PDD	Risperidone	Low	Antipsychotic	Acute, Long	4 weeks, 8 weeks, 24 weeks	Att
Tzitiridou et al.^83^	2005	Greece	NRCT (Within)	70 (45/25)	8.4 (1.2)	Epilepsy	Oxcarbazepine	Low	Antiepileptic	Long	18 months	Att, CF + R, Lan., PM
Werry et al.^84^	1975	New Zealand	RCT (Crossover)	21 (21/0)	8.7 (1.7)	Incontinence	Imipramine	High	Antidepressant	Acute	3 weeks	Att
Wilson and Staton^85^	1984	USA	NRCT (Within)	75 (55/20)	10.8 (5.5–16.0)	MDD	Amitriptyline, Imipramine	High	Antidepressant	Long	> 3 months	Att., CF + R, EF, Int., Lan., PM
Yepes et al.^86^	1977	USA	RCT (Crossover)	22 (21/1)	9.2 (7.3–12.3)	Hyperactivity/aggressive behaviour	Amitriptyline	High	Antidepressant	Acute	2 weeks	Att., EF
Yuan et al.^87^	2018	China	RCT (Between**)	124 (85/39)	6.5 (2.0)	ID	Lithium	Low	Antipsychotic	Long	3 months	Int

*Age reported as mean (SD or range) or median [range].

**Sufficient data available for both within- and between-groups design. Selection was made using protocol outlined in “Methods”.

Studies without description of gender split did not report this information in their original study.

*ADHD* attention deficit hyperactive disorder, *Att.* attention, *BPD* bipolar disorder, *CD* conduct disorder, *CF* + *R* concept formation and reasoning, *Comp.* composite score, *DBD* disruptive behaviour disorder, *EF* executive function, *ID* intellectual disability, *Int.* intelligence, *IQ* intelligence quotient, *Lan.* language, *MDD* major depressive disorder, *Mem.* memory, *NRCT* non-randomized controlled trial, *NR* not reported, *PDD* pervasive developmental disorder, *Perc.* perception, *PM* psychomotor functioning, *RCT* randomized controlled trial, *SAR* seasonal affective rhinitis, *TBI* traumatic brain injury.

### Overall cognition

**Figure 2:**
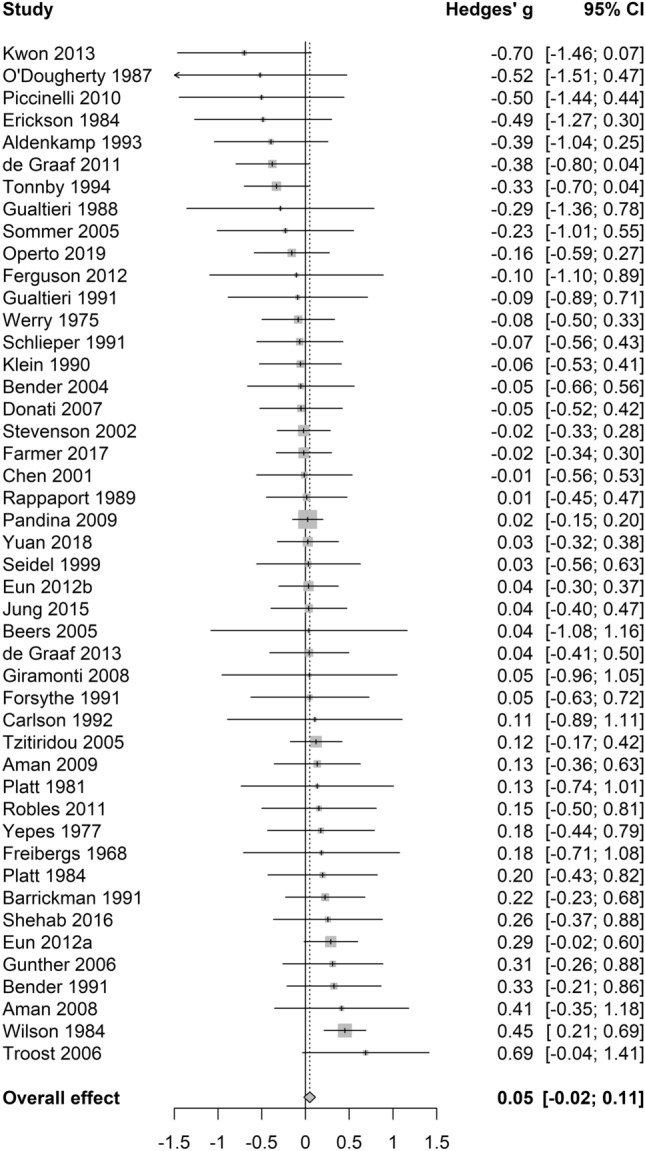
Forest plot for overall cognition analysis.

**Figure 3:**
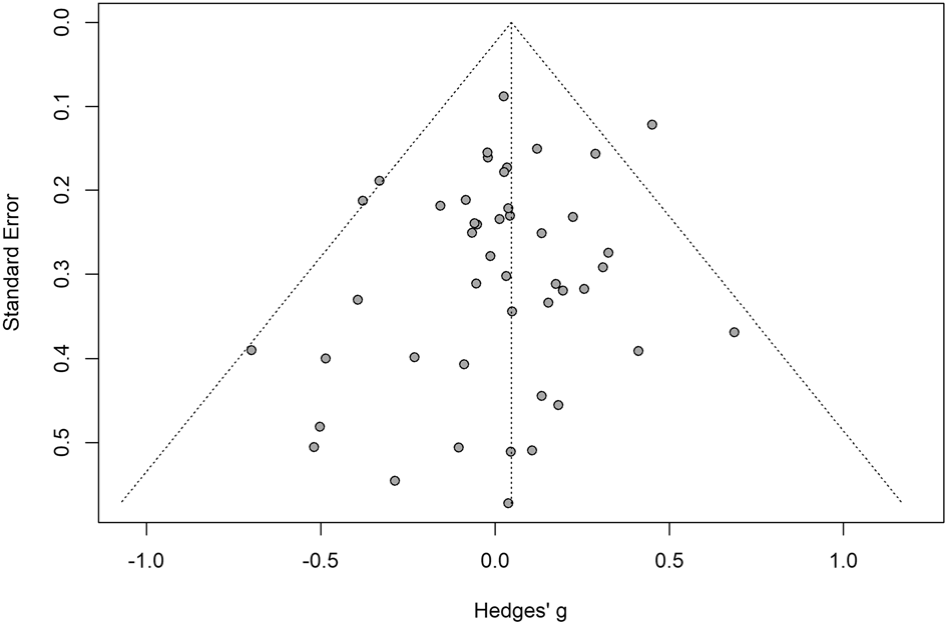
Funnel plot for overall cognition analysis.

Overall, the 46 studies included reported a total of 536 effect sizes. The pooled effect size of the difference between cognition on and off medication across the 46 studies was negligible and non-significant (*g* = 0.05, 95% CI − 0.02 to 0.11, *p* = 0.16; see Fig. 2), with no heterogeneity between studies (τ^2^ = 0, *I*^2^ = 0%, Q = 42.36). The funnel plot did not reveal significant asymmetry (Egger’s intercept = − 0.5, *p* = 0.14; see Fig. 3).

### Subgroup analyses

Pooled estimates for subgroup analyses by anticholinergic drug class, potency, length of administration and cognitive domain are presented in Table 2. The number of studies within individual sub-analyses ranged from 2 to 37. Varying levels of heterogeneity were present across analyses, ranging from null to high (τ^2^ range: 0–0.13, I^2^ range: 0–76.2, Q = 0.18–54.70).

**Table 2:** Pooled estimates for subgroup analyses by anticholinergic drug class, potency, length of administration and cognitive domains.

Subgroup analysis	Pooled estimate				Heterogeneity			Test of between-subgroups differences		
	k	g	95% CI	p value	Tau^2^	I^2^	Q	Q	df	p value
**Drug class**								9.98	5	0.08
Antiepileptic	14	− 0.03	− 0.17–0.11	0.63	0	9.67	14.39			
Antipsychotic	14	0.06	− 0.03–0.16	0.19	0	0	7.44			
Antidepressant	7	0.24	0.01–0.47	0.04	0	13.22	6.91			
Respiratory	5	0.02	− 0.15–0.19	0.75	0	0	1.48			
Opioid analgesic	3	− 0.18	− 0.79–0.44	0.34	0	0	1.84			
Urological	2	− 0.13	− 1.83–1.58	0.52	0	0	0.18			
**Potency**								0.71	1	0.40
Low	36	0.02	− 0.05–0.09	0.50	0	0	27.40			
High	10	0.11	− 0.11–0.33	0.29	0.01	28.02	12.50			
**Length of administration**								2.62	2	0.27
Current and long-term	29	0.07	− 0.03–0.17	0.19	0.01	23.39	36.55			
Current and acute	20	0.05	− 0.04–0.14	0.25	0	0	8.06			
Historical	3	− 0.18	− 0.79–0.44	0.34	0.00	0	1.84			
**Cognitive domain**								5.59	7	0.59
Attention	37	0.04	− 0.04–0.12	0.32	0	0	35.49			
Psychomotor functioning	17	− 0.10	− 0.32–0.11	0.32	0.10	63.24	43.52			
Concept formation and reasoning	13	0.14	− 0.02–0.30	0.08	0.01	15.96	14.28			
Perception	3	0.25	− 0.90–1.39	0.45	0.11	50.18	4.01			
Memory	16	0.04	− 0.06–0.14	0.40	0	0	9.05			
Executive function	15	− 0.01	− 0.27–0.24	0.91	0.12	48.50	27.19			
Intelligence	14	0.08	− 0.18–0.33	0.53	0.13	76.23	54.70			
Language	6	0.11	− 0.07–0.29	0.17	0	0	4.54			

**Figure 4:**
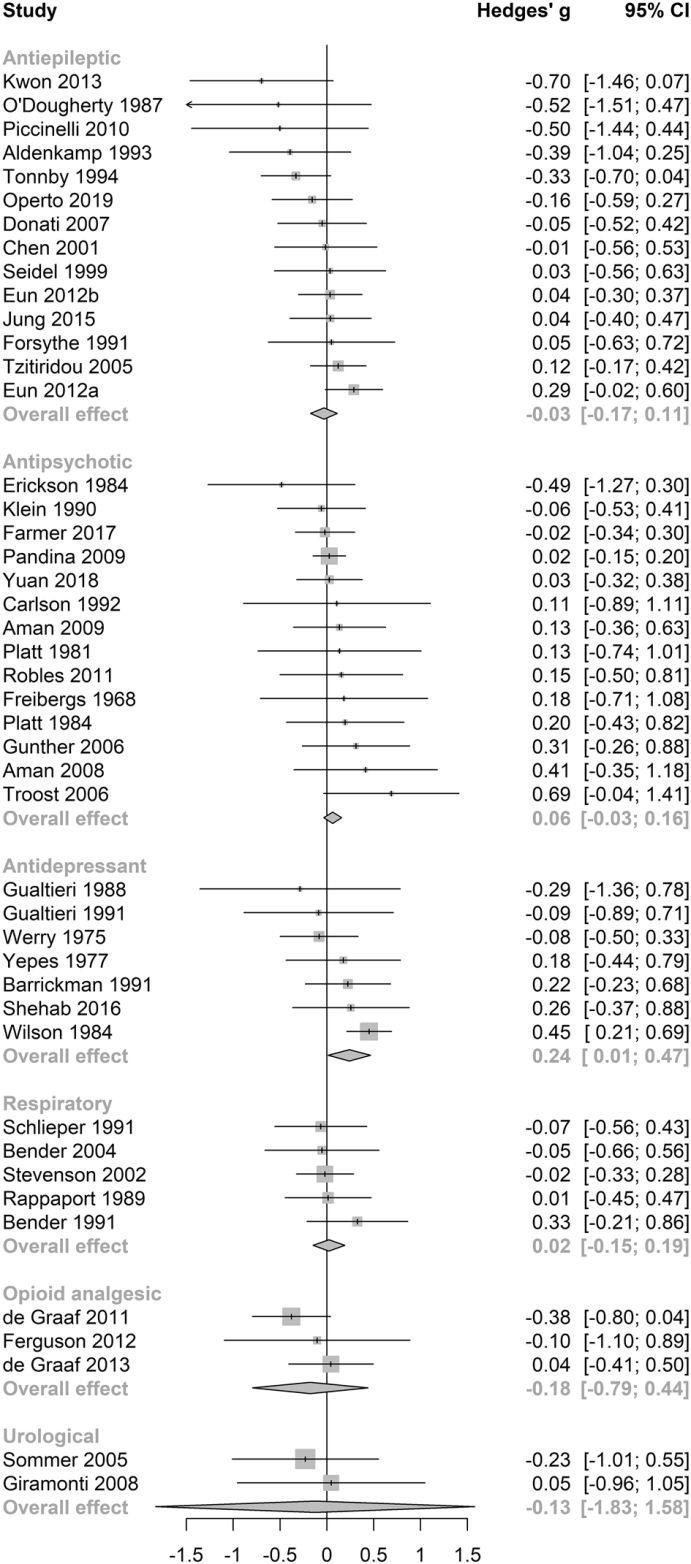
Forest plot for medication class sub-analysis.

**Figure 5:**
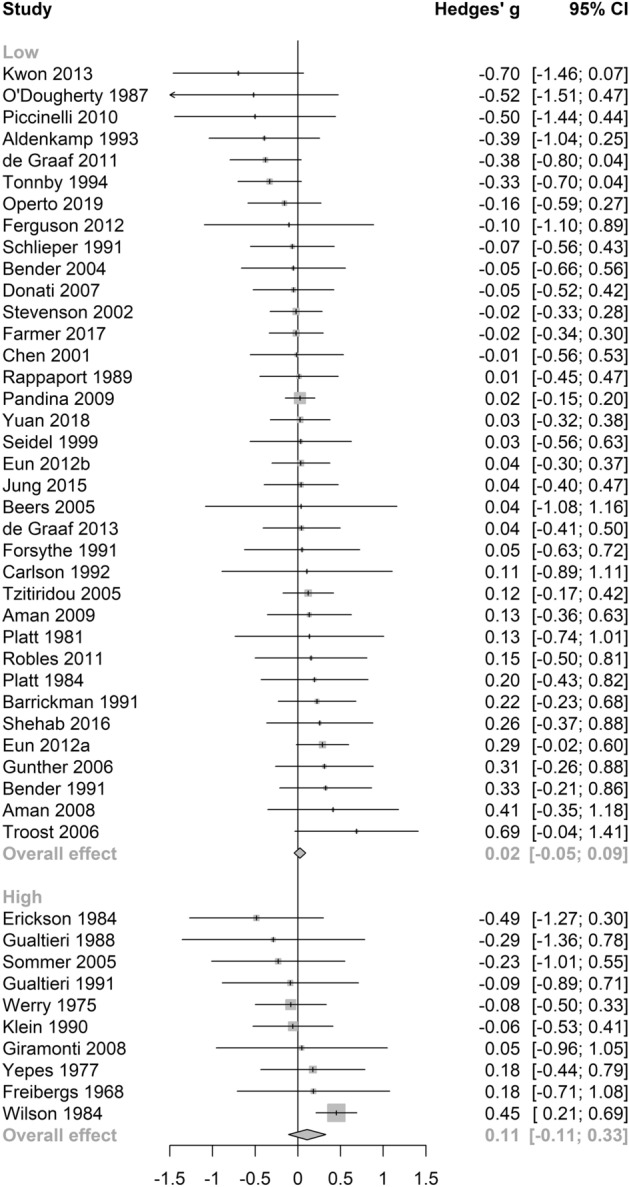
Forest plot for anticholinergic potency sub-analysis.

**Figure 6:**
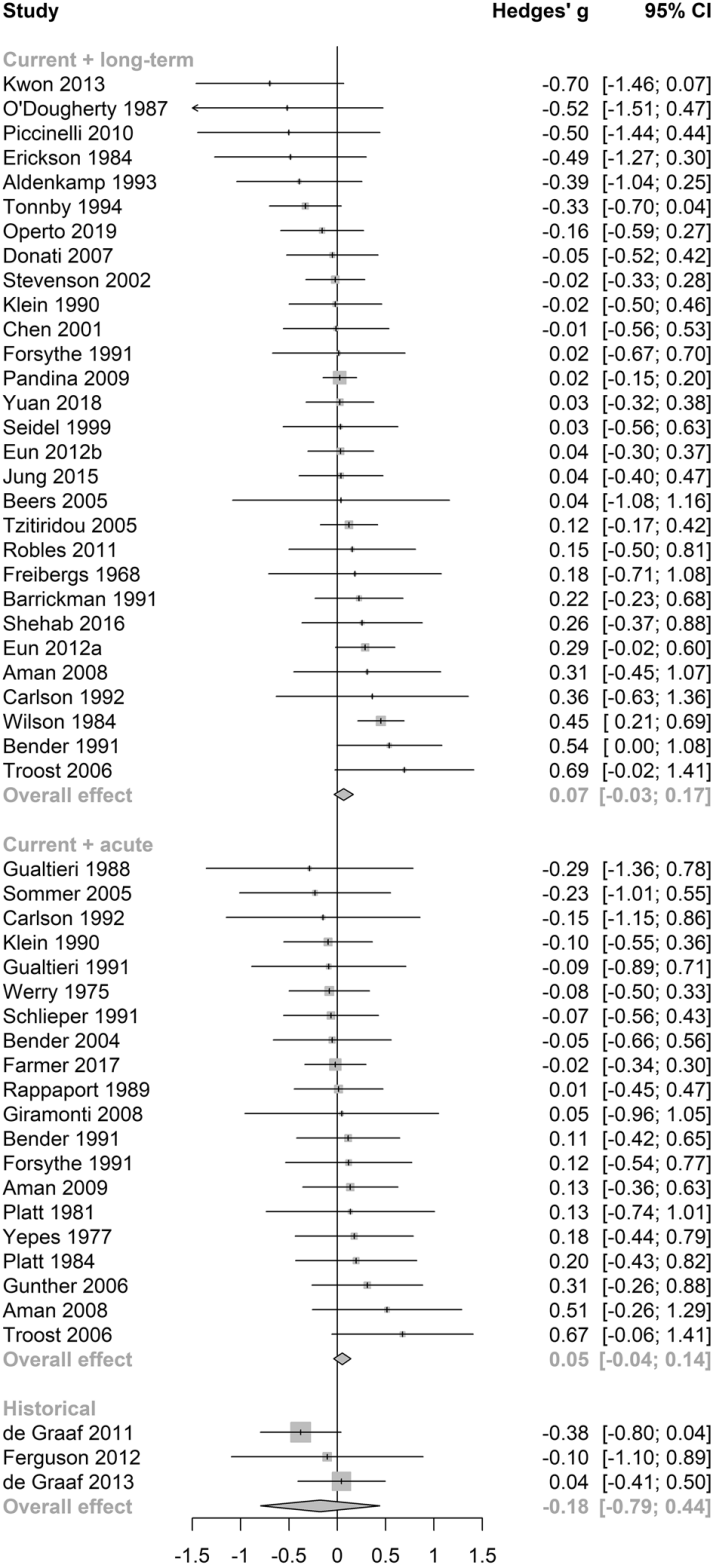
Forest plot for length of administration sub-analysis.

**Figure 7:**
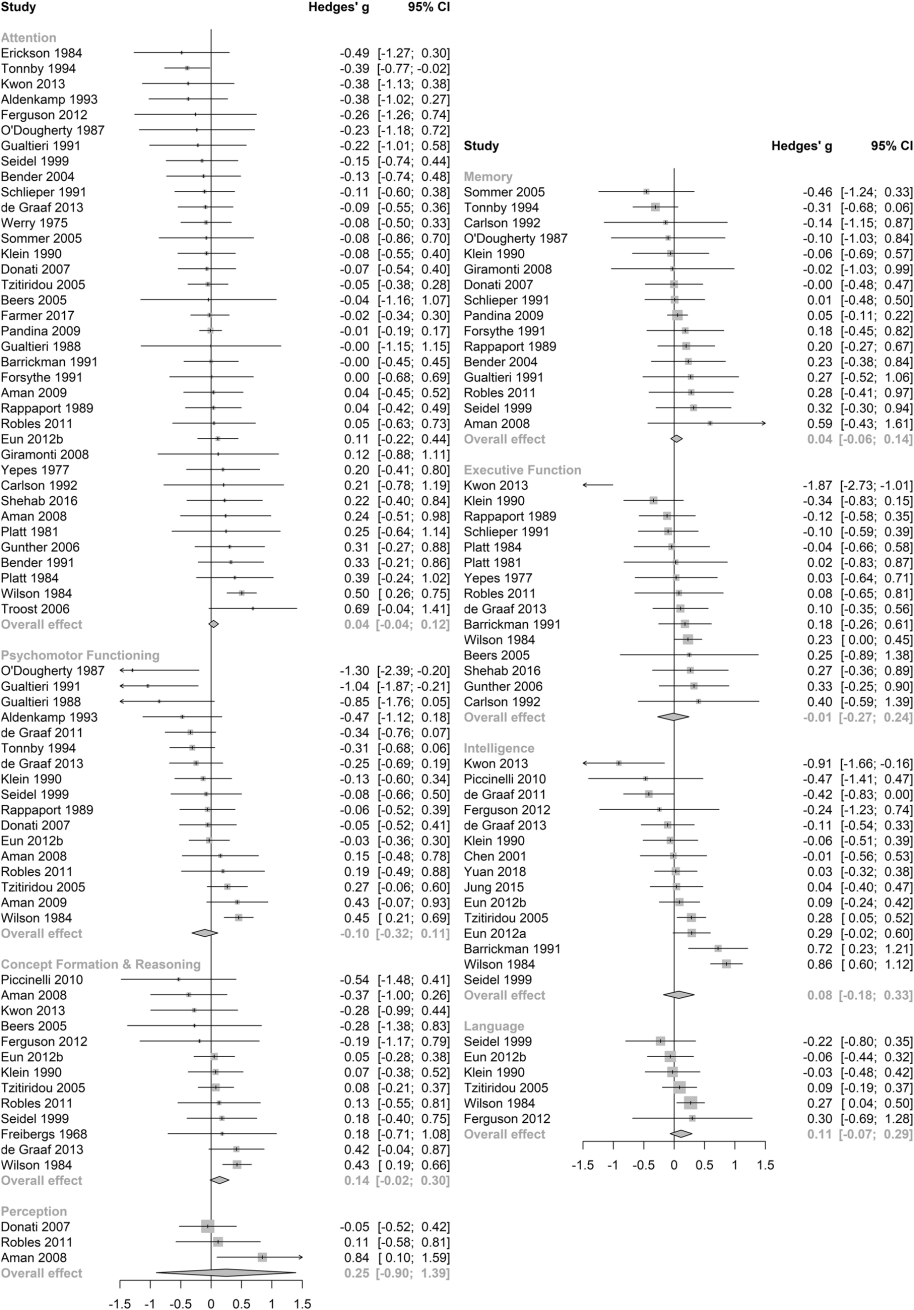
Forest plot for cognitive domain sub-analysis.

No significant differences between subgroups were revealed through a test of between-subgroup differences using the random-effects model (see Table 2). The pooled effect size for cognitive outcomes on antidepressant medications was small and statistically significant (see Table 2 and Fig. 4), with negligible heterogeneity between studies (τ^2^ = 0, *I*^2^ = 13.2%, Q = 6.91). Notably, this effect was not significant in a sensitivity analysis (Supplementary Table 3) which included only studies of high quality. Pooled estimates were non-significant across the remaining anticholinergic drug class (see Fig. 4), potency (see Fig. 5), length of administration (see Fig. 6), and cognitive domain (see Fig. 7) subgroup analyses. All null results were replicated within the sensitivity analysis of high-quality studies, except the memory cognitive domain analysis, which had a small positive significant effect (*g* = 0.09, 95% CI 0.01–0.17, *p* = 0.02).

## Discussion

We quantified the effects of anticholinergic medications on cognition in children systematically across the literature. We report that, unlike older adult samples^7–9^, anticholinergic medications are not associated with cognitive impairments in children. This finding was regardless of the classification approach used: drug class, potency, duration of use, and cognitive domain. The discrepancy between child and older adult samples may be due to shorter lengths of exposure in children, higher rates of polypharmacy in older adults^88^, residual confounding, study design, or biological ageing processes.

Older adults have the opportunity for years or decades of anticholinergic exposure^88^, with polypharmacy common, whereas studies included here from child samples typically had short exposure durations (6 months or less in most studies) and little polypharmacy. It may be that the detrimental effect of anticholinergic medications on cognition in late-adulthood is driven by long exposure and polypharmacy^89,90^, factors not observed in children. Further, in late-life, the class of antidepressant appears to differentially affect cognition, with anti-depressants, urologicals, and antiparkinsonian drugs showing the strongest associations with incident dementia risk^4^. We did not see this pattern of effects in children. It may be that duration of exposure and polypharmacy again drives this difference, however residual confounding in late-life samples cannot be ruled out. It may be that incontinence and mood symptoms, for which anticholinergic medications are prescribed, are early clinical indicators of dementia-related neuropathologies^4^ (which accrue decades prior to a dementia diagnosis^91^) and that early, undiagnosed dementia is driving the associations between use of anticholinergic medicines and poor cognition in adults.

Interestingly, all study designs included in this review were experimental, whereas those included in reviews of older adults are typically longitudinal epidemiological cohort studies^7–9^. Standards of reporting cognitive performance also differ between children and adults. Cognitive performance in children is typically reported as test scores on a continuum, while in adults (especially those in late-life), a dichotomous classification of Neurocognitive Disorders is primarily used (e.g. presence versus absence of mild cognitive impairment or dementia). Study designs and differences in classification of cognition therefore may also underlie differences in the patterns of effects observed in children versus older adults, including the finding that anticholinergic antidepressants displayed a positive association with cognition (albeit with a small effect size, which was not significant when only high-quality studies were included). This small positive effect may be due to the short-term nature of the studies included here and is consistent with a meta-analysis of randomised control trials in adult samples^92^. We do not know the effects of the long-term use anticholinergic antidepressants in children. Notably, a small positive effect of anticholinergic medication on memory was found when only including studies of high quality. Whether this is a true effect, which is counter to that found in adults^93^, needs to be replicated in future studies. Lastly, there are important biological differences between children and adults that would modify the psychopharmacological effects of anticholinergic medications, particularly blood brain permeability^94,95^.

This study is not without limitations. The included studies were biased in terms of geographical representativeness. Fourteen studies were excluded at the full-text stage as they were not in English (of 323) and we do not know if any would have met inclusion criteria; although, given the low number, they would unlikely have changed the conclusions. Authors of papers were contacted, but we either had no response or the author was unable to provide us with the necessary data where it was not presented in text. We assessed the effect of duration of exposure on cognitive outcomes, when total dose or volume of exposure may have been more appropriate. However, this information was inconsistently reported or not reported at all in many of the studies. Therefore, duration of use was used as the best proxy for volume of exposure, with the assumption that longer duration of use would equate to higher volume of exposure. Only 21 of the 100 high- or low-potency anticholinergics identified in a systematic review of anticholinergic medications by Duran et al.^12^ were used in the studies included in this meta-analysis. It may be that different results would be seen had children been exposed to a wider range of anticholinergic medicines. Positively, the vast majority of studies (all but two) utilised valid and reliable cognitive outcome measures, as catalogued specifically or adapted from those detailed in Lezak et al.^96^.

## Conclusion

By pooling effects across previous literature, anticholinergic medications do not appear to detrimentally affect cognitive function in children. In fact, there may be a small positive cognitive benefit of anticholinergic antidepressants, at least in the short-term. Our findings appear to conflict with reviews in older adults, and future studies will have to disentangle the reasons for this.

## Supplementary Information

Supplementary Information.

## Data Availability

All data and code available at https://github.com/ericaghezzi/anticholinergic_med_metaanalysis.
