# Supplementary material for: The effects of anticholinergic medications on cognition in children: a systematic review and meta-analysis
Source: Sci Rep. 2021 Jan 8;11:219. doi: 10.1038/s41598-020-80211-6 (PMC7794471; doi:10.1038/s41598-020-80211-6)
Supplement: Supplementary file 1 — Supplementary Information. [file 41598_2020_80211_MOESM1_ESM.docx]

**The effects of anticholinergic medications on cognition in children:
a systematic review and meta-analysis**

Erica Ghezzi*^1^, Michelle Chan*^1^, Lisa M. Kalisch Ellett^2^, Tyler J. Ross^1^, Kathryn Richardson^3^, Jun Ni Ho^2^, Dayna Copley^1^, Claire Steele^1^, Hannah A.D. Keage^1^

*Equal first authors

^1^Justice and Society, University of South Australia, Adelaide, Australia

^2^Clinical and Health Sciences, University of South Australia, Adelaide, Australia

^3^ Norwich Medical School, University of East Anglia, Norwich, UK

Corresponding author:

#### A/Prof Hannah Keage

University of South Australia

#### GPO BOX 2741

Adelaide

#### South Australia 5000

Hannah.Keage@unisa.edu.au

**Supplementary Materials**

#### **Search Terms**

The search (in pubmed terms) was:

("muscarinic antagonist" OR "muscarinic antagonists" OR "antimuscarinic" OR alimemazine OR baclofen OR bromocriptine OR carbamazepine OR cetirizine OR citalopram OR codeine OR disopyramide OR domperidone OR dosulepin OR entacapone OR fexofenadine OR haloperidol OR hydrocodone OR ketorolac OR lithium OR loperamide OR loratadine OR loxapine OR meperidine OR methadone OR mirtazapine OR molindone OR morphine OR nefazodone OR oxcarbazepine OR phenelzine OR pimozide OR prochlorperazine OR promazine OR risperidone OR tramadol OR trazodone OR amantadine OR chlordiazepoxide OR cimetidine OR clonazepam OR cyclobenzaprine OR diazepam OR digitoxin OR fentanyl OR fluoxetine OR fluvoxamine OR methocarbamol OR olanzapine OR oxycodone OR paroxetine OR propoxyphene OR quetiapine OR fumarate OR ranitidine OR temazepam OR theophylline OR triazolam OR amitriptyline OR atropine OR belladonna OR alkaloids OR benztropine OR chlorphenamine OR chlorpromazine OR clomipramine OR clozapine OR cyproheptadine OR desipramine OR dexchlorpheniramine OR dicyclomine OR diphenhydramine OR doxepin OR fluphenazine OR hydroxyzine OR hyoscyamine OR imipramine OR levomepromazine OR meclozine OR nortriptyline OR orphenadrine OR oxybutynin OR propantheline OR protriptyline OR scopolamine OR hyoscine OR thioridazine OR tolterodine OR trihexyphenidyl OR trimipramine OR "cholinergic antagonist" OR "cholinergic antagonists" OR anticholinergic)

AND (cognit* OR neuropsych* OR learn* OR memory OR "executive function" OR "executive functions")

AND (children OR childhood OR youth* OR teen*)

**Databases:**

- Medline
- PsychInfo
- Embase.

#### **Supplementary Material – Quality Assessment Tool**

Critical Appraisal Tool for RCTs with individual participants in parallel groups.

This checklist and scoring instructions was developed for the purpose of our meta-analysis using a checklist created by the Joanna Briggs Institute.

|  | Yes (1) | No (0) | Unclear (0) | NA (0) |
| --- | --- | --- | --- | --- |
| 1. Was true randomization used for assignment of participants to treatment groups? |  |  |  |  |
| 1. Were participants blind to treatment assignment? |  |  |  |  |
| 1. Were those taking cognitive outcomes (assessors) blind to treatment assignment? |  |  |  |  |
| 1. Was follow up complete and if not, were differences between groups in terms of their follow up adequately described? |  |  |  |  |
| 1. Were participants analyzed in the groups to which they were randomized (i.e. was an intention-to-treat analysis conducted)? |  |  |  |  |
| 1. Were cognitive outcomes reliable and valid? |  |  |  |  |
| 1. Was cognition measured in a standardised way (e.g. procedure consistent; were the assessors adequately trained; if there was more than one assessor, were they similar in ability/experience?). |  |  |  |  |
| 1. Was the trial design appropriate, and any deviations from the standard RCT design (individual randomization, parallel groups) accounted for in the conduct of the trial? |  |  |  |  |
| Notes: | | | | |
|  | Total Score: /8 | | | |

#### **Supplementary Table 1.** PRISMA Checklist

| **Section/topic** | **#** | **Checklist item** | **Reported on page #** |
| --- | --- | --- | --- |
| **TITLE** | | |  |
| Title | 1 | Identify the report as a systematic review, meta-analysis, or both. | p. 1 |
| **ABSTRACT** | | |  |
| Structured summary | 2 | Provide a structured summary including, as applicable: background; objectives; data sources; study eligibility criteria, participants, and interventions; study appraisal and synthesis methods; results; limitations; conclusions and implications of key findings; systematic review registration number. | p. 1 |
| **INTRODUCTION** | | |  |
| Rationale | 3 | Describe the rationale for the review in the context of what is already known. | p. 1-2 |
| Objectives | 4 | Provide an explicit statement of questions being addressed with reference to participants, interventions, comparisons, outcomes, and study design (PICOS). | p. 2 |
| **METHODS** | | |  |
| Protocol and registration | 5 | Indicate if a review protocol exists, if and where it can be accessed (e.g., Web address), and, if available, provide registration information including registration number. | p. 2 |
| Eligibility criteria | 6 | Specify study characteristics (e.g., PICOS, length of follow-up) and report characteristics (e.g., years considered, language, publication status) used as criteria for eligibility, giving rationale. | p. 2 |
| Information sources | 7 | Describe all information sources (e.g., databases with dates of coverage, contact with study authors to identify additional studies) in the search and date last searched. | p. 2 |
| Search | 8 | Present full electronic search strategy for at least one database, including any limits used, such that it could be repeated. | p. 2 (Supp. Materials) |
| Study selection | 9 | State the process for selecting studies (i.e., screening, eligibility, included in systematic review, and, if applicable, included in the meta-analysis). | p. 2 |
| Data collection process | 10 | Describe method of data extraction from reports (e.g., piloted forms, independently, in duplicate) and any processes for obtaining and confirming data from investigators. | p. 2 |
| Data items | 11 | List and define all variables for which data were sought (e.g., PICOS, funding sources) and any assumptions and simplifications made. | p. 2 |
| Risk of bias in individual studies | 12 | Describe methods used for assessing risk of bias of individual studies (including specification of whether this was done at the study or outcome level), and how this information is to be used in any data synthesis. | p. 2 |
| Summary measures | 13 | State the principal summary measures (e.g., risk ratio, difference in means). | p. 3 |
| Synthesis of results | 14 | Describe the methods of handling data and combining results of studies, if done, including measures of consistency (e.g., I^2^) for each meta-analysis. | p. 3 |

*From:*  Moher D, Liberati A, Tetzlaff J, Altman DG, The PRISMA Group (2009). Preferred Reporting Items for Systematic Reviews and Meta-Analyses: The PRISMA Statement. PLoS Med 6(7): e1000097. doi:10.1371/journal.pmed1000097

For more information, visit: **www.prisma-statement.org**.

#### **Supplementary Table 2.** Quality assessment scores for included studies

| **Author** | **Year** | **Quality assessment score (/8)** |
| --- | --- | --- |
| Aldenkamp et al. | 1993 | 4 |
| Aman et al. | 2008 | 6 |
| Aman et al. | 2009 | 7 |
| Barrickman et al. | 1991 | 3 |
| Beers et al. | 2005 | 3 |
| Bender & Milgrom | 2004 | 6 |
| Bender et al. | 1991 | 2 |
| Carlson et al. | 1992 | 4 |
| Chen, Chow, & Lee | 2001 | 5 |
| de Graaf et al. | 2011 | 7 |
| de Graaf et al. | 2013 | 5 |
| Donati et al. | 2007 | 4 |
| Erickson et al. | 1984 | 3 |
| Eun et al. | 2012a | 5 |
| Eun et al. | 2012b | 3 |
| Farmer et al. | 2017 | 6 |
| Ferguson et al. | 2012 | 7 |
| Forsythe et al. | 1991 | 3 |
| Freibergs, Douglas, & Weiss | 1968 | 4 |
| Giramonti, Kogan, & Halpern | 2008 | 5 |
| Gualtieri & Evans | 1988 | 5 |
| Gualtieri, Keenan, & Chandler | 1991 | 5 |
| Gunther et al. | 2006 | 4 |
| Jung et al. | 2015 | 6 |
| Klein | 1990 | 3 |
| Kwon et al. | 2013 | 3 |
| O'Dougherty et al. | 1987 | 3 |
| Operto et al. | 2019 | 2 |
| Pandina, Zhu, & Cornblatt | 2009 | 6 |
| Piccinelli et al. | 2010 | 3 |
| Platt et al. | 1981 | 5 |
| Platt et al. | 1984 | 5 |
| Rappaport et al. | 1989 | 7 |
| Robles et al. | 2011 | 6 |
| Schlieper et al. | 1991 | 6 |
| Seidel & Mitchell | 1999 | 5 |
| Shehab, Brent, & Maalouf | 2016 | 3 |
| Sommer et al. | 2005 | 2 |
| Stevenson et al. | 2002 | 7 |
| Tonnby et al. | 1994 | 3 |
| Troost et al. | 2006 | 3 |
| Tzitiridou et al. | 2005 | 4 |
| Werry, Dowrick, Lampen, & Vamos | 1975 | 6 |
| Wilson & Staton | 1984 | 2 |
| Yepes et al. | 1977 | 5 |
| Yuan et al. | 2018 | 8 |

**Supplementary Table 3.** Results of sensitivity analyses for high-quality studies (rating of 4 or higher on quality assessment scale)

|  | **Pooled Estimate** | | | | **Heterogeneity** | | | **Test of between-subgroups differences** | | |
| --- | --- | --- | --- | --- | --- | --- | --- | --- | --- | --- |
| **Analysis** | **k** | **g** | **95% CI** | **p value** | **Tau^2^** | **I^2^** | **Q** | **Q** | **df** | **p value** |
| Overall cognition | 30 | 0.03 | -0.02 - 0.09 | 0.25 | 0 | 0% | 12.65 |  |  |  |
| **Drug class** |  |  |  |  |  |  |  | 10.45 | 4 | 0.03 |
| Antiepileptic | 7 | 0.09 | -0.08 - 0.25 | 0.25 | 0 | 0 | 4.36 |  |  |  |
| Antipsychotic | 11 | 0.06 | -0.003 - 0.13 | 0.06 | 0 | 0 | 2.44 |  |  |  |
| Antidepressant | 4 | -0.04 | -0.28 - 0.21 | 0.66 | 0 | 0 | 0.74 |  |  |  |
| Respiratory | 4 | -0.03 | -0.08- 0.02 | 0.19 | 0 | 0 | 0.06 |  |  |  |
| Opioid analgesic | 3 | -0.18 | -0.79 - 0.44 | 0.34 | 0 | 0 | 1.84 |  |  |  |
| Urological* | 1 |  |  |  |  |  |  |  |  |  |
| **Potency** |  |  |  |  |  |  |  | 0.44 | 1 | 0.51 |
| Low | 24 | 0.04 | -0.03- 0.10 | 0.25 | 0 | 0 | 11.59 |  |  |  |
| High | 6 | -0.01 | -0.17 - 0.15 | 0.88 | 0 | 0 | 0.96 |  |  |  |
| **Length of administration** |  |  |  |  |  |  |  | 2.59 | 2 | 0.27 |
| Current and long-term | 14 | 0.06 | -0.02 - 0.13 | 0.11 | 0 | 0 | 5.86 |  |  |  |
| Current and acute | 15 | 0.04 | -0.05 - 0.13 | 0.32 | 0 | 0 | 4.27 |  |  |  |
| Historical | 3 | -0.18 | -0.79 - 0.44 | 0.34 | 0 | 0 | 1.84 |  |  |  |
| **Cognitive domain** |  |  |  |  |  |  |  | 8.32 | 7 | 0.31 |
| Attention | 23 | -0.01 | -0.07 - 0.05 | 0.74 | 0 | 0 | 6.87 |  |  |  |
| Psychomotor functioning | 12 | -0.11 | -0.36 - 0.14 | 0.36 | 0.08 | 46.90 | 20.72 |  |  |  |
| Concept formation & reasoning | 7 | 0.11 | -0.10 - 0.32 | 0.26 | 0 | 0 | 4.46 |  |  |  |
| Perception | 3 | 0.25 | -0.90 - 1.39 | 0.45 | 0.11 | 50.18 | 4.01 |  |  |  |
| Memory | 11 | 0.09 | 0.01 - 0.17 | 0.02 | 0 | 0 | 3.02 |  |  |  |
| Executive function | 9 | 0.04 | -0.08 - 0.17 | 0.46 | 0 | 0 | 2.35 |  |  |  |
| Intelligence | 8 | 0.05 | -0.15 - 0.26 | 0.56 | 0.02 | 38.56 | 11.39 |  |  |  |
| Language | 3 | 0.04 | -0.37 - 0.46 | 0.69 | 0 | 0 | 1.19 |  |  |  |

*Insufficient studies for meta-analysis, removed from analysis
